# Non‐Targeted Analysis of Extracellular Vesicle‐Enriched Plasma Proteome Between Early and Late Rugby Playing Career

**DOI:** 10.1002/prca.70036

**Published:** 2025-11-29

**Authors:** Abhishek Jagan, Yusuke Nishimura, Tim Donnovan, Jatin G. Burniston

**Affiliations:** ^1^ Research Institute for Sport & Exercise Sciences Liverpool John Moores University Liverpool UK

**Keywords:** biomarker, muscle injury, neurodegeneration, sports‐related concussion

## Abstract

**Purpose:**

Rugby players experience high‐impact collisions, potentially increasing their risk of neurodegenerative conditions. This study investigates whether the plasma proteome of extracellular vesicles (EV) provides biomarkers to indicate differential risk associated with a rugby career.

**Experimental Design:**

Twenty‐four males were recruited: eight academy rugby players (18 ± 1 years), eight professional rugby players (33 ± 5 years; >10‐year career), and eight CrossFit athletes (32 ± 5 years; no history of collision‐related injuries). EV were enriched from plasma using strong‐anion exchange magnetic microparticles and digested proteins were analyzed by LC‐MS/MS for label‐free quantitation.

**Results:**

A total of 449 proteins were identified (false discovery rate <1%). Statistical analysis on 403 proteins quantified in at least *n* = 3 participants in each group highlighted 52 significant (*p* < 0.05, *q* < 0.01) differences, including 44 proteins that had abundance profiles unique to professional rugby players. Eight proteins which were depleted and three proteins which were elevated have previously recognized roles in neurodegenerative processes.

**Conclusions and Clinical Relevance:**

Proteins associated with neuroprotection were specifically depleted in the plasma EV proteome of long‐serving professional rugby players. The proteins highlighted in professional rugby players could be used to develop biomarker panels for predicting at‐risk athletes or for guiding treatment interventions.

**Summary:**

Repetitive high‐impact collisions experienced by rugby players may predispose them to neurodegenerative conditions, yet the biological processes underpinning this risk remain poorly understood.

This study investigates whether the proteome of plasma extracellular vesicles (EV) could serve as early, minimally invasive biomarkers of neurodegenerative risk in athletes exposed to repeated head impacts.

By comparing the EV proteome of professional rugby players, younger academy athletes, and non‐collision sport controls, we identified specific proteins with known neuroprotective roles that were depleted in long‐serving rugby professionals. These alterations suggest systemic biological changes related to prolonged exposure to collisions.

Our findings provide novel insight by highlighting the potential of EV‐based proteomic profiling as a tool for early detection and monitoring of neurodegeneration‐related processes in at‐risk athletic populations.

This approach could ultimately inform strategies for risk stratification, early intervention, and tailored clinical monitoring in collision sport athletes.

## 1 Introduction

Rugby has a high injury incidence rate compared to other team contact sports and players are regularly exposed to collision injuries, including blunt trauma to the upper body, head and neck. Sport‐related concussion (SRC) is the most common (20% of all match injuries) injury in Rugby Football Union in England [1] and SRC incidence rates are rigorously monitored. The England professional Rugby injury surveillance project [2] reported 18.4 incidences of SRC per 1000 h of professional play during the 2022–23 season. Reliable diagnosis of concussion is the first step in the management of player health but diagnosis can be challenging in the context of team contact sports. The diagnostic criteria for concussion include actual or suspected loss of consciousness [3], which may not always occur and players may also present co‐occurring collision or crush injuries to the torso or neck.

The long‐term consequences of SRC remain to be fully understood but connections have been drawn between concussions and negative consequences on cognitive function, including chronic traumatic encephalopathy (CTE) [4], Alzheimer's disease (AD), and motor neuron disease (MND) [5]. Former elite rugby players may have lower cognitive function compared to non‐contact sport players [6] and older adults with a history of three or more concussions during adulthood have worse cognitive performance than those with no history of concussion [7]. Electroencephalography (EEG) [8], functional magnetic resonance imaging (fMRI) [9], and advanced magnetic resonance imaging (MRI) [10] can each distinguish distinct patterns in individuals with persistent post‐concussion symptoms, and there is growing interest in identifying blood biomarkers for SRC diagnosis.

Plasma biomarker studies in rugby players have targeted biomarkers of neurodegeneration such as Tau, neurofilament light (NFL) [11], glial fibrillar acidic proteins (GFAP) [12], and others [13], as potential indicators of traumatic brain injury (TBI). These hypothesis‐led studies suggest a connection between changes in plasma proteins and the occurrence and recovery of SRC. However, biomarkers, such as S100 Calcium binding protein B (S100B), that are elevated after SRC [14], are also known to be elevated by physical activities such as running [15] and may, therefore, also indicate exercise‐induced muscle damage rather than collision‐specific processes. Plasma proteomics offers broader insights into the physiological responses of athletes [16, 17] and there is growing interest in using omic techniques to discover protein biomarkers related to tissue inflammation and cerebrovascular integrity, particularly in athletes with a history of concussions [18, 19].

Extracellular vesicles (EV), including microvesicles and exosomes, have gained attention for their roles in exercise‐induced cytokine secretion [20] and as mediators of intercellular communication. EV can be characterized by size, cargo, and surface markers and can contain cargoes, including proteins, lipids, metabolites, messenger RNA, micro RNA (miRNA), and nucleic acids [21]. The unique properties of EV, including their resistance to enzymatic degradation, ability to cross the blood‐brain barrier, and traceability to their cell of origin, make them a promising source for TBI biomarkers [22, 23]. Indeed, EV miRNA biomarkers can distinguish between injured and control samples [24] and recent EV proteomic studies have identified proteomic signatures in National Football League (NFL) players at risk of CTE [25].

EV represent a small portion of the plasma proteome and methods for isolating EV, including size exclusion chromatography [26], and ultracentrifugation [27] are labor‐intensive and require specialized equipment. Herein, we employed magnetic polymeric microparticles (Mag‐Net) designed for the isolation, purification, and enrichment of EV from plasma [28]. EV proteomes were studied across three independent groups of athletes, including: young academy rugby players (Acd), older professional rugby players (Pro) with >10 years experience, and CrossFit (Xfit) athletes age‐matched to the Pro players. The Xfit athletes reported no history of impact or concussion injuries and the rugby players had no reported incidence of concussion during the 6 months prior to the collection of samples for this study.

## 2 Methods

### 2.1 Participants

**FIGURE 1:**
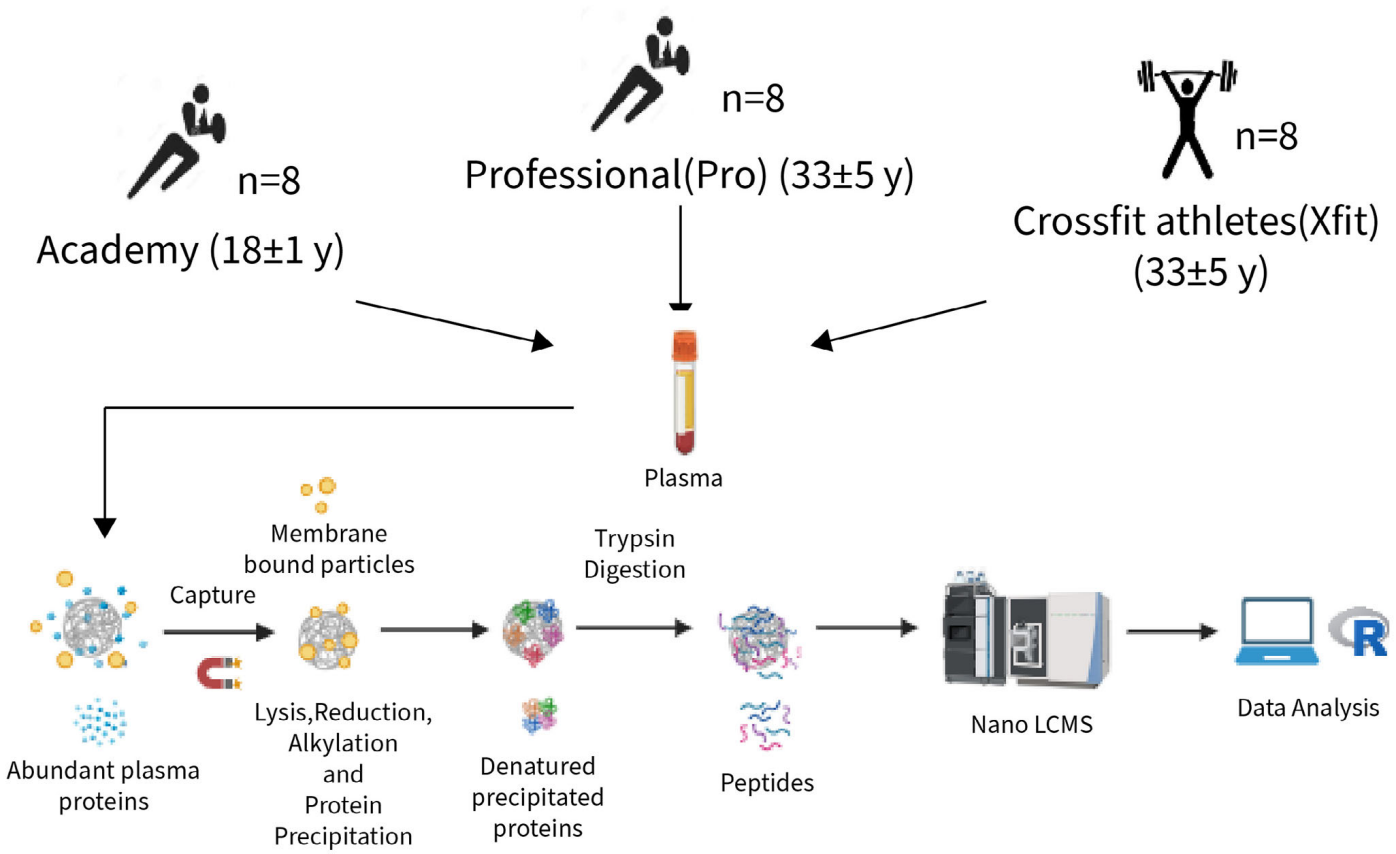
Experimental design and sample processing. Workflow of the quantitative proteomic analysis of the plasma EV isolated using the MagResyn SAX protocol from healthy male Professional (Pro) players (*n* = 8) with 10+ years of professional rugby playing experience, academy (*n* = 8) players in the third year of their youth development program and CrossFit athletes (Xfit) (*n* = 8) age‐matched to professional rugby players with no reported history of concussion or major trauma.

Figure 1A,B provide an overview of the experimental design and analysis protocol. Eight academy players (Acd; 18 ± 1 years) in their third week of preseason with no reported incidence of concussion from the previous 6 months, eight Professional rugby players with 10+ years professional rugby experience (Pro; 33 ± 5 years) in the first week of preseason with no concussions reported for the last 6 months and eight CrossFit athletes (Xfit; 32 ± 5 years) age matched to the Pro group and no history of concussion or other high grade impact injuries, were recruited for blood sampling (Figure 1). All participants were male and gave their informed consent to the procedures approved (M23_SPS_3182) by the Research Ethics Committee of Liverpool John Moores University.

### 2.2 Blood Samples Collection

Blood samples were collected in vacutainers containing Ethylenediaminetetraacetic acid (EDTA). Blood samples were centrifuged at 1200 × *g* for 10 min at 4°C. Plasma was aliquoted and stored at −80°C until further analysis. Freeze/thaw cycles were prohibited.

### 2.3 Membrane Particle Enrichment

Magnetic affinity‐based isolation was used to enrich the membrane particle fraction using a bead‐based protocol developed by Wu et al. [28]. Twenty‐five microliters MagReSyn SAX (Resyn Biosciences, Ranburg, South Africa) (MAG) beads was equilibrated with wash buffer (Wb; 50 mM Bis Tris Propane pH 6.3, 150 mM NaCl) then mixed with 100 µg plasma protein diluted in binding buffer (Bb; 100 mM Bis Tris Propane pH 6.3, 150 mM NaCl) supplemented with cOmplete mini‐ EDTA free protease inhibitor (Roche). During each incubation, the protein and bead slurry was mixed at 550 rpm (ThermoMixer Eppendorf, UK/Ireland). After an initial 30‐min incubation, beads were washed thrice with 500 µL of Wb then incubated in Lysis and Reduction Mix (LR) (50 mM Tris, pH 8.5, 1% SDS, 10 mM TCEP) for 60 min at 37°C with mixing. Iodoacetamide (15 mM final concentration) was added, and samples were incubated protected from light for 30 min with mixing. Beads were washed three times in acetonitrile (ACN) and three times in absolute ethanol prior to on‐bead digestion in 25 mM ammonium bicarbonate and trypsin (1:50 enzyme to protein ratio) at 37°C for 2 h with mixing. Digestion was halted by adding trifluoroacetic acid (TFA) to a final concentration of 0.5 % (v/v). The concentration of the supernatant was estimated using spectrophotometry (260 nm/280 nm ratio; N50 Nanophotometer, IMPLEN, Munich, Germany). Peptide aliquots (5 µg) were purified on C_18_ Zip‐tips (Millipore) and eluted using a 40:60 mix of ACN and 0.1% TFA. Peptide solutions were dried by vacuum centrifugation (Thermo Scientific, SpeedVac SPD1030) for 40 min at 50°C, then reconstituted in 0.1% formic acid for LC‐MS/MS analysis.

### 2.4 Liquid Chromatography‐Mass Spectrometry Analysis

Peptide mixtures were analyzed using an Ultimate 3000 RSLC nano liquid chromatography system (Thermo Scientific) coupled to Q‐Exactive orbitrap mass spectrometer (Thermo Scientific). Samples (700 ng) were loaded on to the trapping column (Thermo Scientific, PepMap 100, 5 µm C18, 300 µm X 5 mm), using ulPickUp injection, for 1 min at a flow rate of 25 µL/min with 0.1% (v/v) TFA and 2% (v/v) ACN. Samples were resolved on a 500 mm analytical column (Easy‐Spray C18 75 µm, 2 µm column) using a gradient of 97.5% A (0.1 % formic acid) 5% B (79.9% ACN, 20% water, 0.1% formic acid) to 70% A: 30% B over 120 min at a flow rate of 300 nL/min. The data‐dependent selection of the top 10 precursors selected from a mass range of *m*/*z* 300–1600 was used for data acquisition, which consisted of a 70,000‐resolution full‐scan MS scan at *m*/*z* 200 (AGC set to 1e6 ions with a maximum fill time of 250 ms). MS/MS data were acquired using quadrupole ion selection with a 3.0 *m*/*z* window, HCD fragmentation with a normalized collision energy of 30 and in the orbitrap analyzer at 17,500‐resolution at *m*/*z* 250 (AGC target 5e4 ion with a maximum fill time of 250 ms). To avoid repeated selection of peptides for MS/MS, the program used a 20s dynamic exclusion window.

### 2.5 Label‐Free Quantitation of Protein Abundances

Progenesis Quantitative Informatics for Proteomics (QI‐P; Nonlinear Dynamics, Waters Corp., Newcastle, UK) was used for label‐free quantitation, consistent with previous studies [29, 30]. Log‐transformed MS data were normalized by inter‐sample abundance ratio, and relative protein abundances were calculated using nonconflicting peptides only. MS/MS spectra were exported in Mascot generic format and searched against the Swiss‐Prot database (2023_08) restricted to “Homo Sapiens (human)” (20,424 sequences) using locally implemented Mascot server (v.2.8; www.matrixscience.com). The enzyme specificity was trypsin with two allowed missed cleavages, carbamidomethylation of cysteine (fixed modification) and oxidation of methionine (variable modification), *m*/*z* error tolerances of 10 ppm for peptide ions and 20 ppm for fragment ion spectra were used. False discovery rate (FDR) was calculated using a decoy database search and only high confidence peptide identifications (>1% FDR) were retained. The Mascot output (xml format) was recombined with MS profile data in Progenesis.

### 2.6 Statistical Analysis

All statistical analyses were performed in R version 4.5.1. Differences between Acd, Pro and Xfit were investigated by comparing the intercepts amongst the linear regression models of each group. Statistical significance was set at *p* < 0.05 and *q*‐values were used to calculate FDR and account for multiple testing. Fuzzy c‐means clustering (Mfuzz package in R) [31] was used to segregate patterns of abundance difference amongst statistically significant proteins. This analysis revealed five distinct cluster patterns, with each protein assigned a membership value (MV) based on its abundance pattern across the three groups.

### 2.7 Bioinformatic Analysis

Gene ontology (GO) and KEGG pathway analyses were performed against whole human genome using ShinyGO (v0.80) [32]. Enrichment of GO terms were accepted at an FDR cutoff 0.005 and pathways were restricted to 10 and a pathway size range of 2–2000. InteractiVenn was used to create Venn diagrams [33].

## 3 Results

### 3.1 Data Reproducibility

The repeatability of the Mag‐NET technique was assessed using two aliquots of a single sample that were processed in parallel to create technical replicates of the processing workflow, encompassing: enrichment, digestion, and LC‐MS/MS analysis. Two hundred and twenty‐four proteins were included in the analysis, Reduced Major Axis (RMA) regression found a significant (*R* ^2^ = 0.665, *p* < 0.0001) linear relationship between replicates (Figure S1A). The 95% confidence interval (CI) of the intercept and slope were used to assess fixed‐ and proportional‐bias, respectively. The 95% CI for the Intercept (−1.4390 to 2.2910) did span zero (i.e., no fixed bias was detected), whereas the 95% CI for the slope (0.8201–0.8766) did not span 1, which suggests proportional bias existed between replicate analyses. Coefficient of variation was used to assess the technical variation of protein‐specific data The mean CV was 38.1% (SD 30.6%) and the median and inter‐quartile range in CV was M = 29.7% (Q1 16.5% to Q3 53.3%) (Figure S1B).

### 3.2 EV Proteome Validation

**FIGURE 2:**
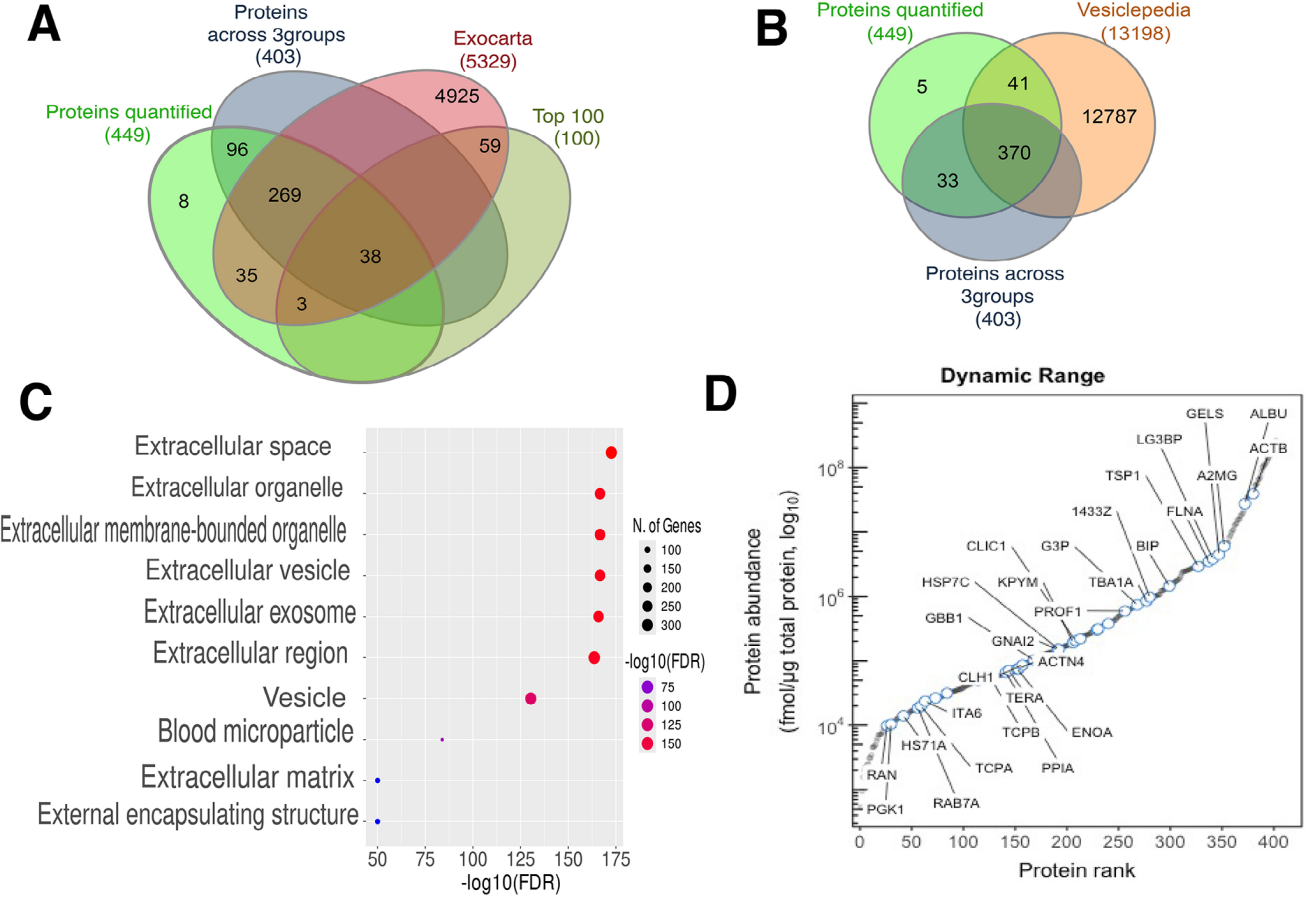
MagResyn enrichment of Plasma EV proteins. Venn diagrams illustrate overlap between proteins in the current study and annotated EV databases: (A) Exocarta and Top 100 exosomes and (B) Vesiclepedia. (C) Gene ontology—Cellular component enrichment analysis of current study against whole human genome. (D) Abundance distribution of 403 proteins ranked (left to right) from lowest to highest abundance. Protein annotations (UniProt accession ids) represent 38 proteins from the top 100 exosomes of the Exocarta database detected in the current study.

In total, 449 proteins were confidently identified (>1 unique peptide at FDR <1%) in plasma EV samples, 411 of which were curated in the Vesiclepedia [34] database and 344 were curated Exocarta [35] database (Figure 2A,B). Of the 344 proteins that overlapped with Exocarta database, 41 proteins were amongst the top 100 most abundant proteins in exosomes (Figure 2B). After filtering to remove missing values, 403 proteins with a minimum of *n* = 3 samples per group were used to investigate statistical differences between Pro, Acd, and Xfit groups.

The dynamic range of protein abundances included in the statistical analysis spanned from 0.002 to 663.02 nmol/µg total protein and encompassed 38 proteins from the top 100 exosome proteins reported in Exocarta (Figure 2D). The high abundance proteins included chaperones (heat shock proteins; HS71A, HSP7C, BIP), actin and actin binding proteins (ACTB, FLNA, ACTN4) and proteins of metabolism and transport (ENOA, GBB1, G3P, KPYN, CLIC1) as well as glycoproteins, protein kinase C inhibitors (1433F, 1433Z, TSP1) and ubiquitin related protein (UBA1) (Table S1).

**FIGURE 3:**
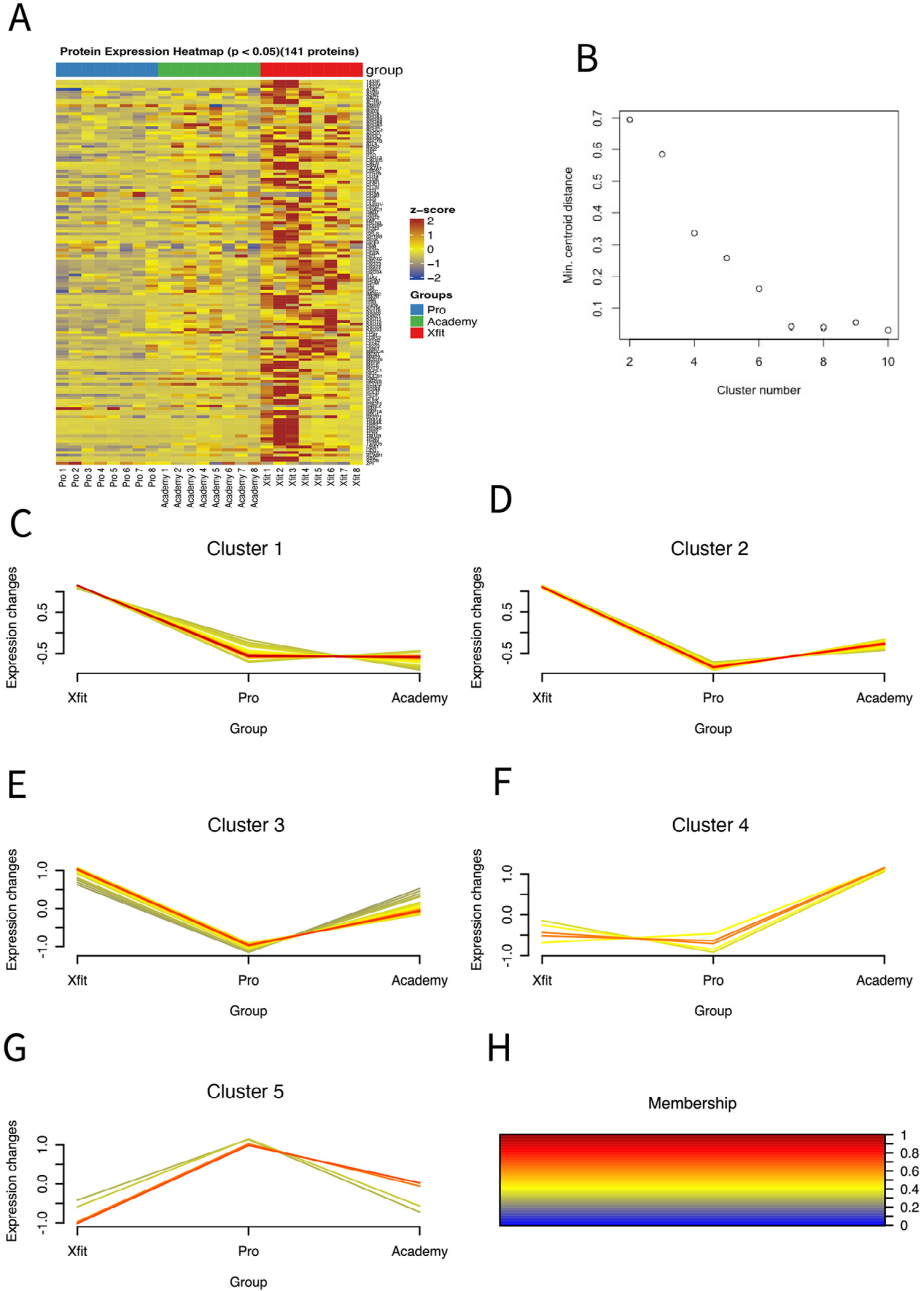
Pattern analysis of protein abundance differences between groups. (A) Heatmap representing the abundance pattern of 52 proteins that exhibited statistically significant differences (One‐way ANOVA; *p* < 0.05, *q* < 0.01). (B) Scree plot used to determine the appropriate number of clusters prior to Fuzzy c‐means clustering on 141 significantly (*p* < 0.05) different proteins. (C‐H) Clusters (1–5) illustrating patterns of protein abundance differences between Academy, Pro and Xfit athletes with no history in contact sports. (F) Color palette representing the membership value of proteins in each cluster. (Membership reflects the degree of similarity of each protein to the cluster centroid) [Xfit = CrossFit athletes; Pro = Professional rugby players; Academy = Academy rugby players].

Statistical analysis highlighted 141 significant (*p* < 0.05) differences in protein abundance between Acd, Pro, and Xfit groups (Figure 3A) and 52 of these proteins had a false discovery rate (FDR) < 1% (*p* < 0.05 and *q* < 0.01). Gene ontology cellular components (GO:CC) enriched amongst the significant proteins included Extracellular space [enrichment FDR (eFDR) = 9.8E−63], Extracellular region (eFDR = 7.2E−63), and Extracellular organelle (eFDR = 8.5E−49) (Figure 2C). Fuzzy c‐means clustering segregated the 141 proteins significant at *p* < 0.05 into five patterns of abundance difference amongst Acd, Pro, and Xfit groups. All abundance measurements, statistical outcomes (*p*‐ and *q*‐values) and assignments to the numbered clusters are reported in Table S2.

Cluster 1 comprised 65 proteins that were specifically more abundant in older athletes without a history of contact sport (Xfit) and were enriched in GO:BP including Platelet aggregation (eFDR = 3.39E−16) and Platelet Activation (eFDR = 3.65E−16), and KEGG pathways such as Motor protein (eFDR = 1.24E−08) and Cytoskeleton in muscle cells (eFDR = 1.82E−05).

Clusters 2 and 3 encompassed 34 and 31 proteins, respectively, and were most abundant in Xfit athletes, intermediate in Acd players and had the lowest abundance in Pro rugby players. Functional enrichment analysis of Cluster 2 proteins included GO: BP terms involving Complement Activation, classical pathway (eFDR = 6.40E−05) and Regulation of triglyceride catabolic process (eFDR = 0.0003) and KEGG pathway associated to Complement and coagulation cascades (eFDR = 1.38E−09) and Cholesterol metabolism (eFDR = 0.0441). Functional enrichment analysis of Cluster 3 proteins highlighted enrichment in GO: BP involving Immunoglobulin mediated immune response (eFDR = 0.00883) and B cell mediated immunity (eFDR = 0.00883) and KEGG pathways associated with Lipid and atherosclerosis (eFDR = 0.0214) and Vitamin digestion and absorption (eFDR = 0.0269).

Cluster 4 included six proteins that were less abundant in Pro rugby athletes compared to Acd and Xfit groups and was enriched in GO: BP terms, including Lipid transport (eFDR = 0.0297) and Lipid localization (eFDR = 0.0297) and KEGG pathways associated with Cholesterol metabolism (eFDR = 0.0598) and PPAR signaling pathway (eFDR = 0.0598).

Cluster 5 consisted of five proteins (AMBP, C4A, C4B, RAN, SERPINA10), that were more abundant in Pro rugby players only, and were associated with GO: BP terms, regulation of apoptotic cell clearance (eFDR = 0.0003) and Complement Activation, classical pathway (eFDR = 0.0020) and KEGG pathways associated with Pertussis (eFDR = 0.0001) and Complement and coagulation cascades (eFDR = 0.0010). All Cluster annotations, including enrichment FDR, fold enrichment and GO Pathway enrichment are reported in Tables S2 and S3.

**FIGURE 4:**
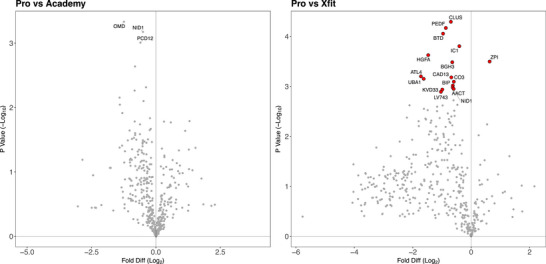
Effect of age and rugby career on protein expression. Visual representation of statistical differences between groups. (A) Volcano plot illustrating log_2_ fold‐difference between Pro/Academy, statistically significant differences (*p* < 0.05) are annotated with UniProt identifiers. (B) Volcano plot illustrating log_2_ fold‐difference between Pro/ Xfit, statistically significant differences (*p* < 0.05, *q* < 0.01) are highlighted in red and annotated with UniProt identifiers. [Xfit = CrossFit athletes; Pro = Professional rugby players; Academy = Academy rugby players; Fold Diff = Fold difference].

Volcano plots were used to illustrate protein‐specific detail relating to the contrasts between pairs of experimental groups (Figure 4). Forty‐six proteins exhibited differences (*p* < 0.05) between Pro and Acd groups (Figure 4A) but only four proteins (OMD, NID1, PCD12, UFO) had an FDR < 20% (*p* < 0.05, *q* < 0.2) and no proteins were significantly different at the 1% FDR criterion used in the three‐way analysis (Figure 3A) of Pro, Acd, and Xfit groups. Differences between Pro and Xfit groups were more numerous and encompassed 114 differences (*p* < 0.05), including 16 proteins (CLUS, PEDF, BTD, IC1, HGFA, ZPI, BGH, ATL4, CAD13, UBA1, CO3, BIP, NID1, AACT, KVD33, LV743) at <1% FDR (Figure 4B).

### 3.3 Analysis of Proteins Significantly Different Only in Pro Rugby Players

**FIGURE 5:**
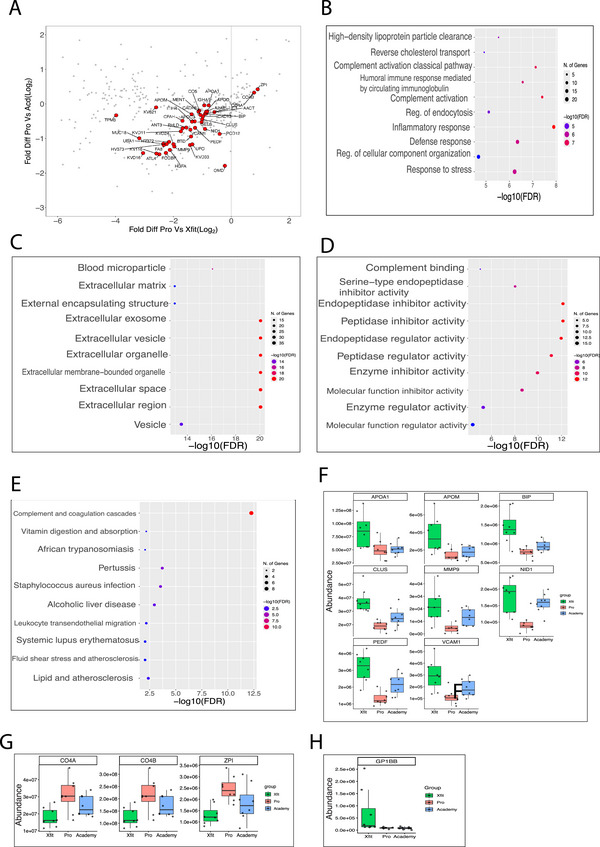
Proteins significantly different in Pro rugby players. (A) Scatterplot comparing protein abundance log_2_ fold‐difference between Pro/ Xfit (x‐axis) and Pro/Academy (y‐axis). Statistically significant (*p* < 0.05, *q* < 0.01) proteins are coloured red and annotated with UniProt identifiers. (B to E) Functional enrichment analysis of 21 proteins from lower left quadrant of scatterplot, including biological process (B), Cellular component (C), Molecular Function (D) and KEGG pathway (E). (F) Box plots of eight proteins specifically less abundant in Pro group. (G) Box plots of three proteins specifically more abundant in Pro group. (H) Box plot of 1 protein enriched in brain reported in the Human Protein Atlas. [Xfit = CrossFit athletes; Pro = Professional rugby players; Academy = Academy rugby players; Fold Diff. = Fold difference].

Figure 5A, illustrates proteins that were significantly different (*p* < 0.05, *q *< 0.01) in samples from Pro rugby players compared to either aged‐matched athletes with no history of contact sports (Xfit group) or younger academy (Acd) rugby players. Proteins (*n* = 52) that exhibited significant differences (one way ANOVA; *p* < 0.05; *q* < 0.01) amongst the three experimental groups were plotted based on their fold difference between either Pro rugby players versus age‐matched Xfit athletes with no history in contact sports (x‐axis) or Pro versus Acd rugby players (y‐axis). The majority of proteins occupy the bottom left quadrant and were less abundant in older rugby players than either age‐matched Xfit athletes or Academy rugby players. The bottom left quadrant included 42 proteins, which shared GO: BP terms including High‐density lipoprotein particle remodeling and clearance (eFDR = 7.89E−05), Lipoprotein metabolic process (eFDR = 7.89E−05) and Reverse cholesterol transport (eFDR = 7.89E−05) and (Table S5).

## 4 Discussion

The risk of neurological damage to competitors in contact sports including rugby is a serious concern and currently little is known about the mechanisms that may predispose athletes with long careers in contact sports to a greater risk of neurological disease. Plasma biomarkers offer an opportunity to objectively assess biological differences and, in particular, non‐targeted analysis of the EV proteome may generate new insight into processes involved in neurological disorders. Eight of the proteins (APOA1, APOM, CLUS, BIP, VCAM1, NID1, MMP9, and PEDF) that were depleted and three proteins (CO4A, CO4B, and ZPI) that were elevated in EV samples from Pro rugby players have previously recognized roles in neurodegenerative processes. Lower levels of the depleted proteins have been associated with various neurological conditions, and their shared response in Pro rugby players could be the basis of a biomarker panel for monitoring the accumulative consequences of sports injuries.

Low levels of apolipoprotein A‐I (APOA1) correlate with greater risk of ischemic strokes and neurodegeneration, and lower serum APOA1 levels may contribute to metabolic dysregulation and neuroinflammation following concussions [36]. Similarly, apolipoprotein M (APOM), which plays a crucial role in the anti‐inflammatory properties of high‐density lipoprotein (HDL) [37], has been linked to impaired endothelial function and increased inflammation when APOM levels are reduced [38]. PDEF is a neurotrophic and neuroprotective protein that declines with age and exhibits an inverse correlation with amyloid‐beta (Aβ) plaques in AD patients [39]. Clusterin (CLUS), also known as apolipoprotein J (APOJ), is an extracellular chaperone that is strongly associated with AD [40]. CLUS promotes the clearance of toxic Aβ peptides [41], and variants in the CLUS gene are an acknowledged marker of late onset AD [42]. CLUS also has neuroprotective roles during acute events such as perioperative ischemic‐reperfusion injury [43], which suggests CLUS deficiency could exacerbate neuronal damage following concussive sports injuries.

The complement system is implicated in neurological disorders, including AD, and complement proteins are a known cargo of plasma EV [44]. Fifteen complement proteins were detected in the current analysis and both A and B fragments of complement protein 4, which are involved in activation of the classical complement pathway, were more abundant in Pro participants. In mice, experimental TBI is associated with long‐term increases in the expression of complement activation (C2, C3 and C4) [45] and anti‐complement agents can promote recovery of experimental TBI [46].

The endoplasmic reticulum chaperone (BiP), also known as GRP78, is critical for intracellular stress responses, and the endoplasmic reticulum is also intimately connected with EV formation [49]. BiP may be particularly relevant during recovery from sports‐related concussions when the brain experiences metabolic disturbances and increases in oxidative stress. BiP expression is increased in experimental models of TBI [50], whereas lower levels of BiP are associated with greater susceptibility to cellular stress and may contribute to conditions such as AD [51]. BiP is also exposed on the outer membrane of brain microvascular endothelia cells and autoantibodies to GRP78 modulate blood brain barrier (BBB) function [52].

Differences in the abundance of brain‐enriched proteins in plasma can give insight into changes in BBB integrity [47] but only one protein, platelet glycoprotein Ib beta chain (GP1BB), of the 52 significant proteins (Figure 3A), is listed amongst the brain enriched transcripts in the Human Protein Atlas [48]. GP1BB is a cell membrane protein that mediates platelet adhesion and was significantly more abundant in Xfit compared to both Acd or Pro rugby players (Figure 5H). On the other hand, vascular cell adhesion molecule 1 (VCAM1), which is an adhesion molecule protein expressed on endothelial cells and is involved in maintaining BBB integrity was less abundant in Pro samples. In the chronic cerebral hypoperfusion model of vascular dementia, VCAM1 expression is increased in brain endothelia cells and correlates with BBB dysfunction [53]. Plasma levels of VCAM1 correlate strongly with age in humans and mice, and brain endothelia cells exposed to plasma from aged mice upregulate VCAM1 abundance [54]. This suggests low circulating levels of VCAM1 may be beneficial, potentially limiting excessive inflammation and maintaining BBB integrity, which is critical for neuronal health.

The vascular basement membrane is a component of the BBB [55] and two proteins, Nidogen‐1 (NID1) and matrix metalloproteinase 9 (MMP9), that were depleted in samples from Pro rugby players are associated with the basement membrane. NID1 is a key structural component of the basement membrane that interlinks with and stabilizes laminin and collagen IV, and is crucial for neuronal health and function [56]. NID1 interacts with reelin which is a basement membrane protein that was more abundant in plasma EV of former NFL players [25]. Matrix metalloproteinase 9 (MMP9), is also implicated in the stabilization and maintenance of the extracellular matrix (ECM) and patients with AD exhibit lower levels of plasma MMP9 [57].

Recently, plasma EV have emerged as a cascading multisystem physiological adaptation that trigger immune responses [58]. Circulating EV have also been shown to exhibit anti‐inflammatory effects in response to varied training regimes [59] and exercise‐induced muscle damage is associated with changes to plasma EV profile [60]. One protein, Z‐dependent protease inhibitor (ZPI), also known as SERPINA10, was elevated in Pro samples. ZPI is known to form a complex with Protein Z (PZ), which enhances the ability of PZ to inhibit coagulation factors, including factor Xa and factor Xia [61]. The actions of ZPI prevent excessive thrombin generation during the early stages of coagulation [62] and reduce thrombotic risk. ZPI may also have protective roles in endothelial injury by preventing oxidized low‐density lipoprotein (ox‐LDL)‐mediated endothelial injury and inhibiting processes, including endothelial‐to‐mesenchymal transition, inflammation, and apoptosis [63].

In this study, we employed the Mag‐Net method [28] that relies on both size‐based “netting” and charge‐based binding to enrich membrane bound particles, including EV from blood plasma samples. We compared our dataset to manually curated EV databases (Exocarta and Vesiclepedia) and confirmed the selective enrichment of EV proteins. Nevertheless, a good proportion of the putative biomarkers highlighted in our analysis of Pro rugby samples were apolipoproteins that are more likely associated with lipid particles such as HDL. Lipid particles are more abundant in plasma than EV and the protein composition of lipid particles and EV differ. Apolipoprotein APOA1, which is a marker of HDL and chylomicron particles, is co‐extracted by traditional EV isolation workflows that rely on size exclusion chromatography [64]. Wu et al. [28] reports lipoproteins are depleted ∼80% compared to raw plasma using the Mag‐Net protocol and, specifically, APOA1 was −2.5 (log2) less abundant in enriched samples. Nevertheless, the apolipoprotein biomarkers highlighted in the current work may not be of EV origin.

Because EV are able to traverse the blood–brain barrier, research on SRC has explored plasma EV as a potential source of biomarkers [18]. However, it is important to clarify our study did not collect data on the incidence or type of injuries experienced by our participants. We also cannot rule out the contribution of musculoskeletal injuries, which occur frequently in Rugby, or lifestyle factors that could affect the plasma EV profile. Future studies are required to verify whether the biomarker pattern discovered herein persists when physical activity level, recent injury history and sample collection protocols are controlled. Nevertheless, despite samples from Acd and Pro players being collected in different pre‐season phases and the lack of control over the time between sample collection and the last bout of exercise along with limited dietary control, we were able to detect consistent differences in the plasma EV proteome.

## 5 Conclusion

Non‐targeted analysis highlighted that proteins associated with neuroprotection were specifically depleted in the plasma EV proteome of long‐serving professional rugby players compared to younger academy rugby players or age‐matched cross‐fit athletes that did not have a history of collision‐related sports injuries. Our findings bring new insight into the biological processes affected by a professional rugby playing career but further studies are required to test whether the putative biomarkers reported herein are linked to sports‐related concussion and musculoskeletal injuries.

## Funding

The authors have nothing to report.

## Conflicts of Interest

The authors declare no conflicts of interest

## Supporting information




**Supporting File 1**: prca70036‐sup‐0001‐Figure S1.pdf.

**Supporting File 2**: prca70036‐sup‐0002‐Table S1.xlsx.

**Supporting File 3**: prca70036‐sup‐0003‐Table S2.xlsx.

**Supporting File 4**: prca70036‐sup‐0004‐Table S3.xlsx.

**Supporting File 5**: prca70036‐sup‐0005‐Table S4.xlsx.

**Supporting File 6**: prca70036‐sup‐0006‐Table S5.xlsx.

## Data Availability

The mass spectrometry proteomics data have been deposited to the ProteomeXchange Consortium via the PRIDE [1] partner repository with the dataset identifier PXD061593 an https://doi.org/10.6019/PXD061593.
